# Hémangiome surrénalien: à propos d’un cas

**DOI:** 10.11604/pamj.2017.28.172.8299

**Published:** 2017-10-24

**Authors:** Noureddine Njoumi, Nabil Jakhlal, Mohammed Laaroussi, Faicel Mohafid, Mohammed Najih, Hicham Iraki, Aziz Zentar

**Affiliations:** 1 Service de Chirurgie Viscérale II, Hôpital Militaire d’Instruction Mohammed V, Rabat, Maroc

**Keywords:** Hémangiome, glande surrénale, hémangiome caverneux, laparoscopie, Hemangioma, adrenal gland, cavernous hemangioma, laparoscopy

## Abstract

L’hémangiome surrénalien est une entité histologique très rare appartenant au groupe des incidentalomes. Il est souvent asymptomatique, de découverte fortuite lors d’un examen d’imagerie sollicité par une autre affection abdominale. Nous rapportons dans ce travail un seul cas permettant d’étoffer la série internationale qui reste jusqu’à nos jours très limitée.

## Abstract (en)

Adrenal gland hemangioma is a very rare histologic entity belonging to the group of incidentalomas. It is often asymptomatic, unexpectedly detected during imaging examination and resulting from another abdominal affection. We here report a single case to further enrich international case series which are still very limited.

## Introduction

L’hémangiome surrénalien (HS) est une tumeur vasculaire bénigne de la glande surrénale. Cette localisation très rare n’a été rapportée qu’une soixantaine de fois dans la littérature. Son aspect radiologique est non spécifique. Le diagnostic de certitude est histologique après résection chirurgicale qui reste le seul traitement disponible. Nous rapportons un nouveau cas d’HS et discutons parallèlement ses différents aspects épidémiologique, diagnostique et thérapeutique.

## Patient et observation

**Figure 1:**
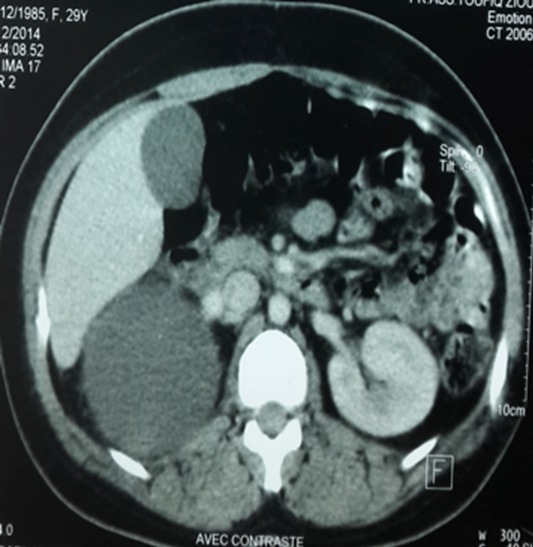
TDM abdominale injectée montrant l’hémangiome surrénalien droit

**Figure 2:**
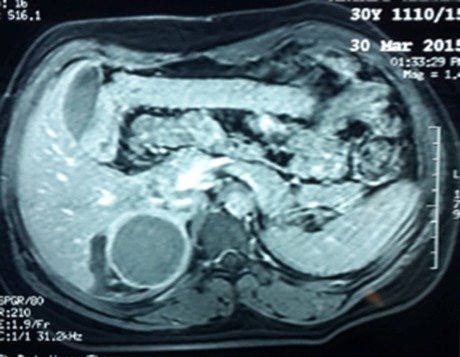
IRM abdominale de l’hémangiome surrénalien droit

**Figure 3:**
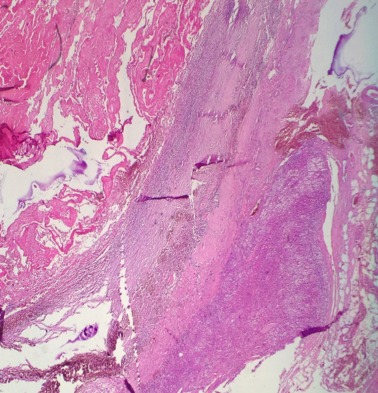
Parenchyme surrénalien siège d’une prolifération tumorale bien limitée faite des structures vasculaires (G×25)

**Figure 4:**
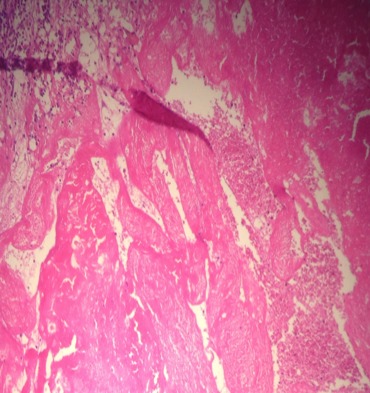
Vaisseaux de taille variable d’aspect fontomique, siège d’une nécrose ischémique (G×400)

Il s’agit d’une patiente de 30 ans, sans antécédents pathologiques notables, qui a été admise initialement pour prise en charge d’une tumeur kystique du foie. La patiente rapportait depuis quatre mois des douleurs intermittentes de l’hypochondre droit avec sensation de pesanteur. L’examen abdominal et général à l’admission était sans particularité. L’échographie abdominale a mis en évidence une image kystique de 7 cm de diamètre de la face inférieur du segment VI du foie en rapport probablement avec un kyste hydatique. Ces données ont été relativement confirmées par un complément scannographique quoi que son aspect radiologique n’était pas typique évoquant des doutes sur une localisation surrénalienne (Figure 1). Effectivement, une imagerie par résonance magnétique réalisée a objectivé une masse surrénalienne droite, de signal tissulaire hétérogène, rehaussée en périphérie de façon annulaire fine et mesurant 7 cm (Figure 2). Devant la taille de la tumeur qui était supérieure à 6 cm, la résection chirurgicale s’est imposée et l’étude anatomopathologique de la pièce opératoire a conclu à un HS remanié avec des zones de nécrose (Figure 3 et Figure 4). Les suites opératoires étaient simples.

## Discussion

L’hémangiome surrénalien est une tumeur rare, bénigne, et non sécrétante [1]. Depuis le premier cas en 1955, seuls 63 cas d’HS ont été rapportés dans les différentes bases de données [2]. Il est, la plupart du temps, de type caverneux et unilatérale, apparaissant entre 50 et 70 ans, et prédominant deux fois plus fréquemment chez la femme [1, 3, 4]. Cependant, quelques cas d’HS chez des patients plus jeunes ont été rapportés comme notre patiente [2, 3]. L’HS est le plus souvent une tumeur non fonctionnelle, et seulement trois cas d'HS sécrétants ont été signalés jusqu’à présent; deux entre eux montrant un excès des minéralocorticoïdes et l’autre un excès de glucocorticoïdes [5, 6]. Les patients sont généralement asymptomatiques. Cependant, les grandes masses peuvent être palpables et parfois spontanément rompues entrainant un choc hypovolémique [7]. Bien que l'échographie ne soit pas utile pour différencier l’HS des autres lésions surrénaliennes, la tomodensitométrie (TDM) et l'imagerie par résonance magnétique (IRM) semblent être contributives. Le principal aspect scannographique d’HS comprend une lésion hypodense, hétérogène avec des calcifications. Ces dernières ont été signalées dans 28-87% des cas et elles correspondent à des phlébolithes qui se répartissent dans toute la zone tumorale [8]. Cependant, ces calcifications manquent de spécificité vu que le carcinome corticosurrénalien, l'hémorragie, la tuberculose et le mélanome métastatique peuvent également les présenter [8]. Le principal diagnostic différentiel est le corticosurrénalome notamment en cas de wash-out retardé sur le scanner injecté. L'IRM montre un signal de faible intensité sur les images en T1 et un hypersignal en T2. Ces lésions sont généralement bien encapsulées et situées dans le cortex surrénal. Lors de l’examen histopathologique, la plupart des tumeurs signalées étaient de type caverneux et rarement capillaire. En plus, elles peuvent subir des modifications dégénératives comme la thrombose, l’hémorragie, la nécrose et les calcifications. Les HS sont considérées comme des masses en rapport avec une éctasie des canaux sinusoïdaux engorgés de sang, qui ont érodé et refoulé les tissus avoisinants. En outre, la présence de multiples cavités vasculaires à la périphérie est une caractéristique importante et responsable d’un rehaussement nodulaire périphérique au scanner injecté [5]. La résection chirurgicale reste nécessaire pour les volumineuses masses surrénales excédant 4cm [9], même lor'squ’elles sont soupçonnées dêtre de nature angiomateuse en raison de la tendance à saigner et de l'incapacité de se prononcer sur les éléments de la malignité. Plusieurs voies d’abord d’exérèse tumorale en chirurgie ouverte ont été décrites, à savoir les approches antérieure, latérale et thoraco-abdominale. La surrénalectomie laparoscopique est devenue la procédure de choix pour des lésions de petite taille ne dépassant pas 6cm [4]. Des résections laparoscopiques pour des masses plus volumineuses allant jusqu'à 12cm de diamètre, ont été rapportées [10].

## Conclusion

L'hémangiome surrénalien est une pathologie rare, qu’on ne doit pas omettre devant une masse surrénalienne, notamment en présence de calcifications à l’imagerie. Le diagnostic positif n’est obtenu qu’après analyse anatomopathologique de la pièce d’exérèse. Le traitement approprié est l’ablation chirurgicale.

## Conflits d’intérêts

Les auteurs ne déclarent aucun conflit d'intérêt.
